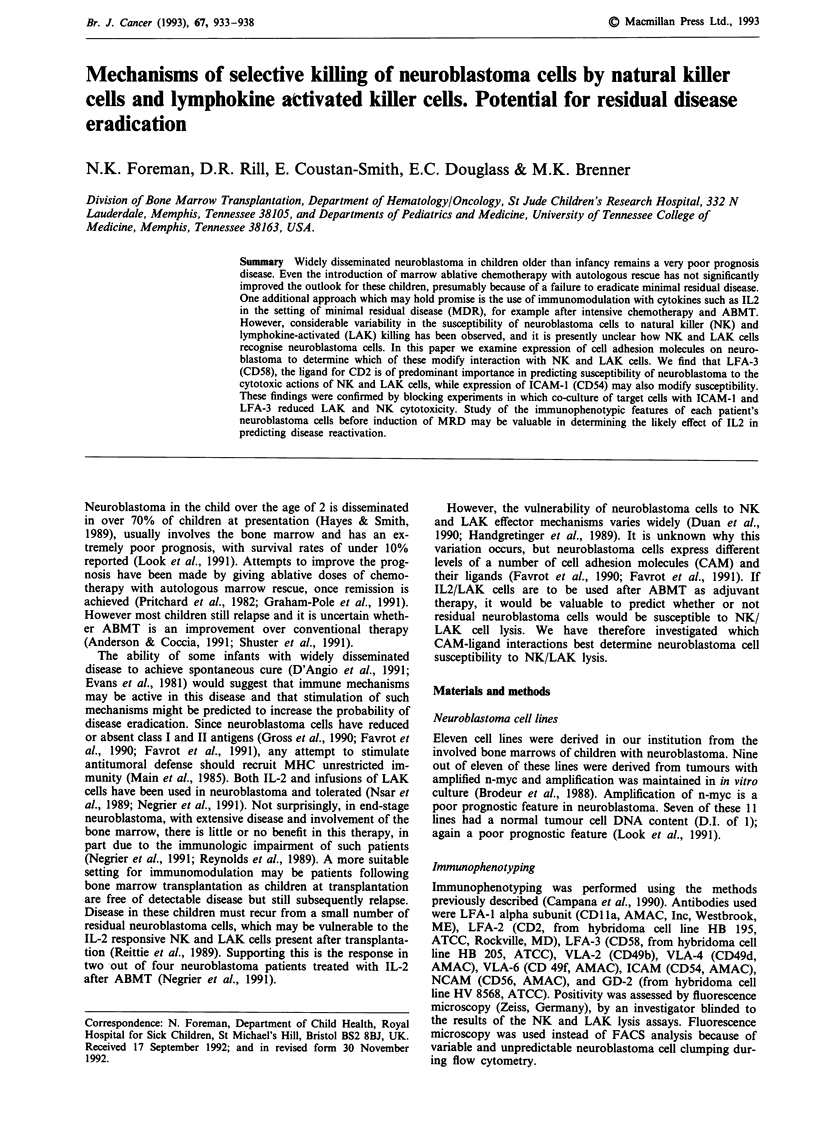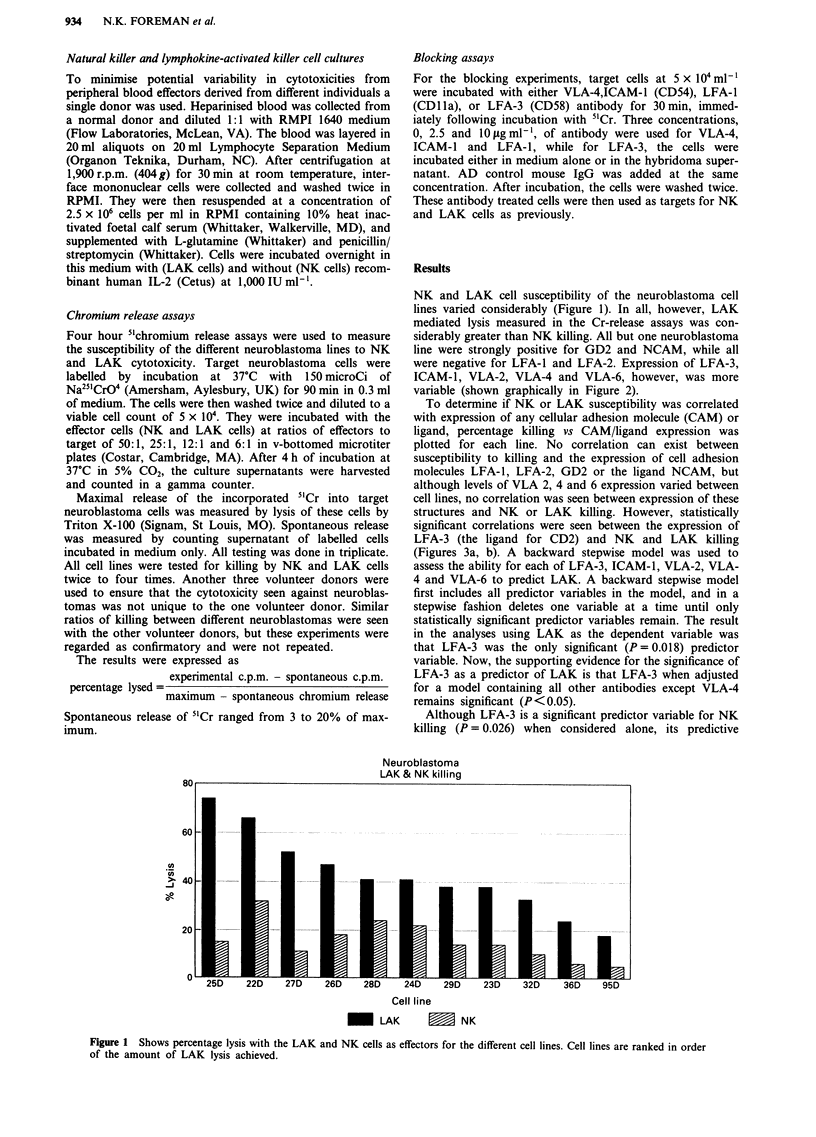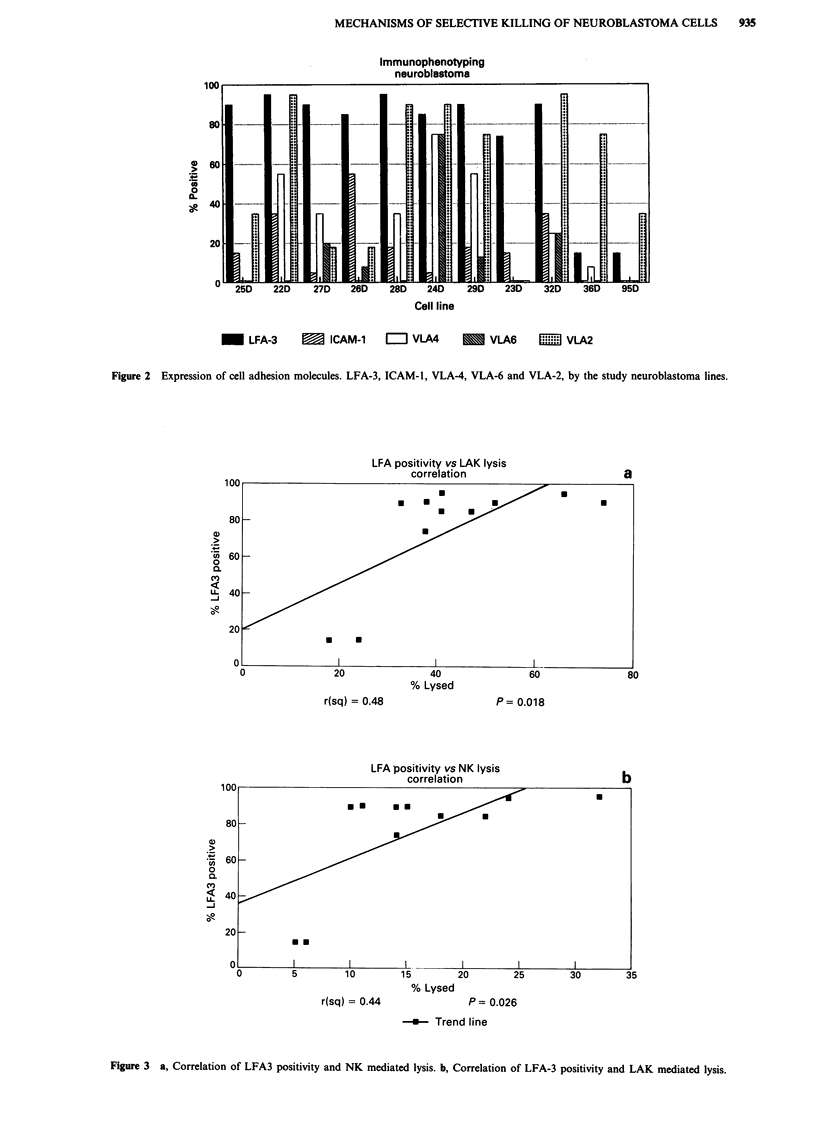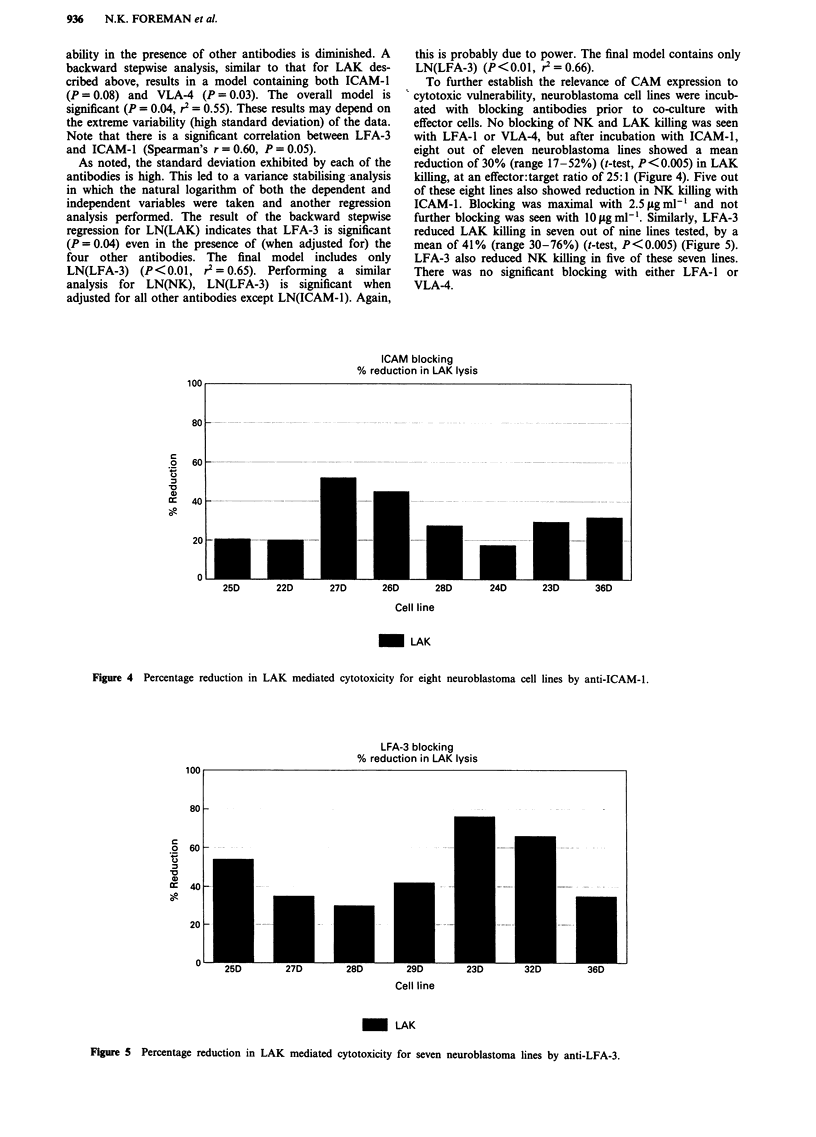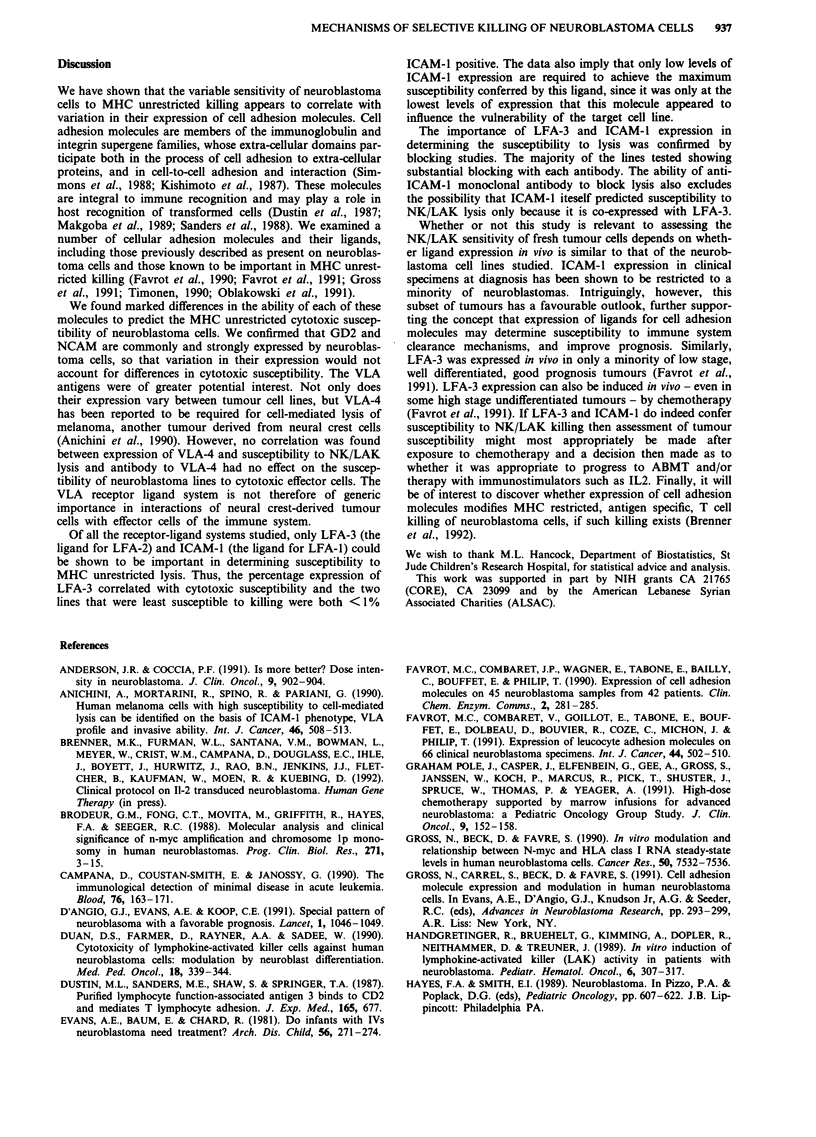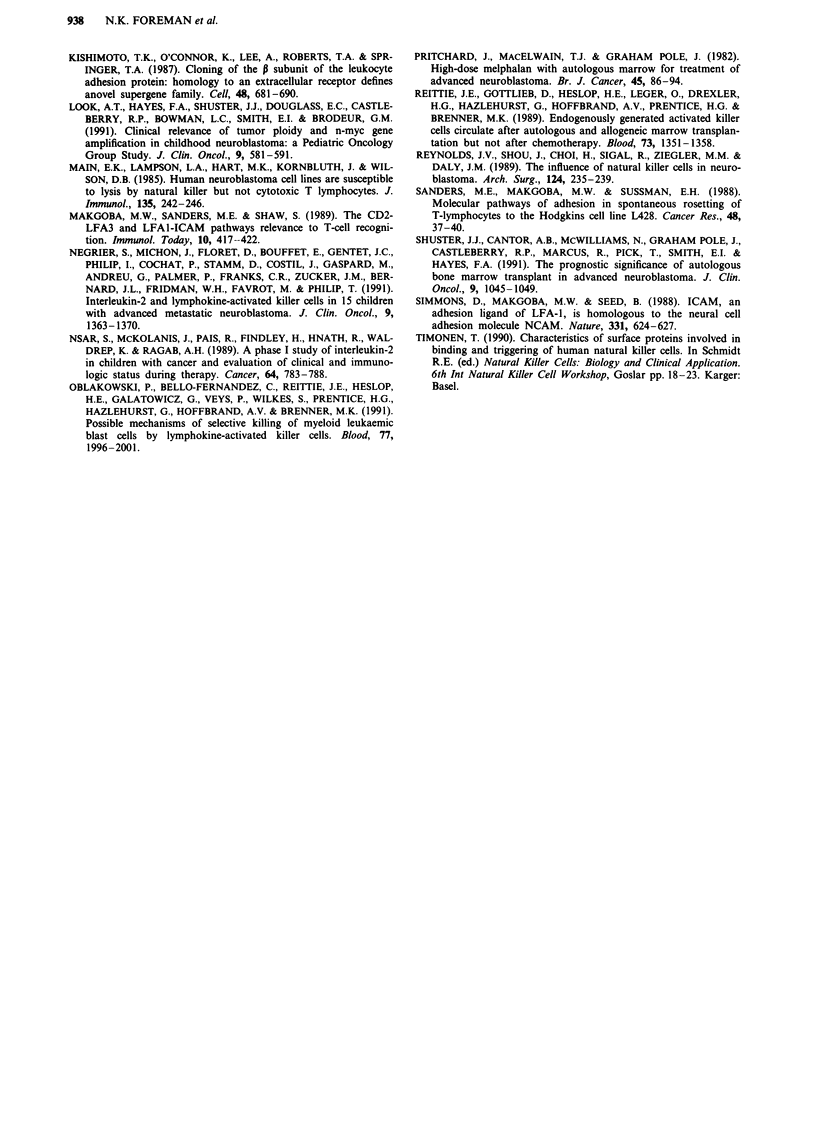# Mechanisms of selective killing of neuroblastoma cells by natural killer cells and lymphokine activated killer cells. Potential for residual disease eradication.

**DOI:** 10.1038/bjc.1993.173

**Published:** 1993-05

**Authors:** N. K. Foreman, D. R. Rill, E. Coustan-Smith, E. C. Douglass, M. K. Brenner

**Affiliations:** Department of Hematology/Oncology, St Jude Children's Research Hospital, Memphis, Tennessee 38105.

## Abstract

Widely disseminated neuroblastoma in children older than infancy remains a very poor prognosis disease. Even the introduction of marrow ablative chemotherapy with autologous rescue has not significantly improved the outlook for these children, presumably because of a failure to eradicate minimal residual disease. One additional approach which may hold promise is the use of immunomodulation with cytokines such as IL2 in the setting of minimal residual disease (MDR), for example after intensive chemotherapy and ABMT. However, considerable variability in the susceptibility of neuroblastoma cells to natural killer (NK) and lymphokine-activated (LAK) killing has been observed, and it is presently unclear how NK and LAK cells recognise neuroblastoma cells. In this paper we examine expression of cell adhesion molecules on neuroblastoma to determine which of these modify interaction with NK and LAK cells. We find that LFA-3 (CD58), the ligand for CD2 is of predominant importance in predicting susceptibility of neuroblastoma to the cytotoxic actions of NK and LAK cells, while expression of ICAM-1 (CD54) may also modify susceptibility. These findings were confirmed by blocking experiments in which co-culture of target cells with ICAM-1 and LFA-3 reduced LAK and NK cytotoxicity. Study of the immunophenotypic features of each patient's neuroblastoma cells before induction of MRD may be valuable in determining the likely effect of IL2 in predicting disease reactivation.


Br. J. Cancer (1993), 67, 933-938 © Macmillan Press Ltd., 1993

### Mechanisms of selective killing of neuroblastoma cells by natural killer cells and lymphokine activated killer cells. Potential for residual disease eradication

N.K. Foreman, D.R. Rill, E. Coustan-Smith, E.C. Douglass & M.K. Brenner

*Division of Bone Marrow Transplantation, Department of Hematology/Oncology, St Jude Children's Research Hospital, 332 N Lauderdale, Memphis, Tennessee 38105, and Departments of Pediatrics and Medicine, University of Tennessee College of Medicine, Memphis, Tennessee 38163, USA.*

**Summary** Widely disseminated neuroblastoma in children older than infancy remains a very poor prognosis disease. Even the introduction of marrow ablative chemotherapy with autologous rescue has not significantly improved the outlook for these children, presumably because of a failure to eradicate minimal residual disease. One additional approach which may hold promise is the use of immunomodulation with cytokines such as IL2 in the setting of minimal residual disease (MDR), for example after intensive chemotherapy and ABMT. However, considerable variability in the susceptibility of neuroblastoma cells to natural killer (NK) and lymphokine-activated (LAK) killing has been observed, and it is presently unclear how NK and LAK cells recognise neuroblastoma cells. In this paper we examine expression of cell adhesion molecules on neuroblastoma to determine which of these modify interaction with NK and LAK cells. We find that LFA-3 (CD58), the ligand for CD2 is of predominant importance in predicting susceptibility of neuroblastoma to the cytotoxic actions of NK and LAK cells, while expression of ICAM-1 (CD54) may also modify susceptibility. These findings were confirmed by blocking experiments in which co-culture of target cells with ICAM-1 and LFA-3 reduced LAK and NK cytotoxicity. Study of the immunophenotypic features of each patient's neuroblastoma cells before induction of MRD may be valuable in determining the likely effect of IL2 in predicting disease reactivation.

Neuroblastoma in the child over the age of 2 is disseminated in over 70% of children at presentation (Hayes & Smith, 1989), usually involves the bone marrow and has an extremely poor prognosis, with survival rates of under 10% reported (Look *et al.*, 1991). Attempts to improve the prognosis have been made by giving ablative doses of chemotherapy with autologous marrow rescue, once remission is achieved (Pritchard *et al.*, 1982; Graham-Pole *et al.*, 1991). However most children still relapse and it is uncertain whether ABMT is an improvement over conventional therapy (Anderson & Coccia, 1991; Shuster *et al.*, 1991).

The ability of some infants with widely disseminated disease to achieve spontaneous cure (D'Angio *et al.*, 1991; Evans *et al.*, 1981) would suggest that immune mechanisms may be active in this disease and that stimulation of such mechanisms might be predicted to increase the probability of disease eradication. Since neuroblastoma cells have reduced or absent class I and II antigens (Gross *et al.*, 1990; Favrot *et al.*, 1990; Favrot *et al.*, 1991), any attempt to stimulate antitumoral defense should recruit MHC unrestricted immunity (Main *et al.*, 1985). Both IL-2 and infusions of LAK cells have been used in neuroblastoma and tolerated (Nsar *et al.*, 1989; Negrier *et al.*, 1991). Not surprisingly, in end-stage neuroblastoma, with extensive disease and involvement of the bone marrow, there is little or no benefit in this therapy, in part due to the immunologic impairment of such patients (Negrier *et al.*, 1991; Reynolds *et al.*, 1989). A more suitable setting for immunomodulation may be patients following bone marrow transplantation as children at transplantation are free of detectable disease but still subsequently relapse. Disease in these children must recur from a small number of residual neuroblastoma cells, which may be vulnerable to the IL-2 responsive NK and LAK cells present after transplantation (Reittie *et al.*, 1989). Supporting this is the response in two out of four neuroblastoma patients treated with IL-2 after ABMT (Negrier *et al.*, 1991).

Correspondence: N. Foreman, Department of Child Health, Royal Hospital for Sick Children, St Michael's Hill, Bristol BS2 8BJ, UK.
Received 17 September 1992; and in revised form 30 November 1992.

However, the vulnerability of neuroblastoma cells to NK and LAK effector mechanisms varies widely (Duan *et al.*, 1990; Handgretinger *et al.*, 1989). It is unknown why this variation occurs, but neuroblastoma cells express different levels of a number of cell adhesion molecules (CAM) and their ligands (Favrot *et al.*, 1990; Favrot *et al.*, 1991). If IL2/LAK cells are to be used after ABMT as adjuvant therapy, it would be valuable to predict whether or not residual neuroblastoma cells would be susceptible to NK/LAK cell lysis. We have therefore investigated which CAM-ligand interactions best determine neuroblastoma cell susceptibility to NK/LAK lysis.

### Materials and methods

### *Neuroblastoma cell lines*

Eleven cell lines were derived in our institution from the involved bone marrows of children with neuroblastoma. Nine out of eleven of these lines were derived from tumours with amplified n-myc and amplification was maintained in *in vitro* culture (Brodeur *et al.*, 1988). Amplification of n-myc is a poor prognostic feature in neuroblastoma. Seven of these 11 lines had a normal tumour cell DNA content (D.I. of 1); again a poor prognostic feature (Look *et al.*, 1991).

### *Immunophenotyping*

Immunophenotyping was performed using the methods previously described (Campana *et al.*, 1990). Antibodies used were LFA-1 alpha subunit (CD11a, AMAC, Inc, Westbrook, ME), LFA-2 (CD2, from hybridoma cell line HB 195, ATCC, Rockville, MD), LFA-3 (CD58, from hybridoma cell line HB 205, ATCC), VLA-2 (CD49b), VLA-4 (CD49d, AMAC), VLA-6 (CD 49f, AMAC), ICAM (CD54, AMAC), NCAM (CD56, AMAC), and GD-2 (from hybridoma cell line HV 8568, ATCC). Positivity was assessed by fluorescence microscopy (Zeiss, Germany), by an investigator blinded to the results of the NK and LAK lysis assays. Fluorescence microscopy was used instead of FACS analysis because of variable and unpredictable neuroblastoma cell clumping during flow cytometry.

*Natural killer and lymphokine-activated killer cell cultures*

To minimise potential variability in cytotoxicities from peripheral blood effectors derived from different individuals a single donor was used. Heparinised blood was collected from a normal donor and diluted 1:1 with RMPI 1640 medium (Flow Laboratories, McLean, VA). The blood was layered in 20 ml aliquots on 20 ml Lymphocyte Separation Medium (Organon Teknika, Durham, NC). After centrifugation at 1,900 r.p.m. (404 *g*) for 30 min at room temperature, interface mononuclear cells were collected and washed twice in RPMI. They were then resuspended at a concentration of $2.5 \times 10^6$ cells per ml in RPMI containing 10% heat inactivated foetal calf serum (Whittaker, Walkerville, MD), and supplemented with L-glutamine (Whittaker) and penicillin/streptomycin (Whittaker). Cells were incubated overnight in this medium with (LAK cells) and without (NK cells) recombinant human IL-2 (Cetus) at 1,000 IU $ml^{-1}$.

*Chromium release assays*

Four hour $^{51}$chromium release assays were used to measure the susceptibility of the different neuroblastoma lines to NK and LAK cytotoxicity. Target neuroblastoma cells were labelled by incubation at 37°C with 150 microCi of $Na_2{}^{51}CrO_4$ (Amersham, Aylesbury, UK) for 90 min in 0.3 ml of medium. The cells were then washed twice and diluted to a viable cell count of $5 \times 10^4$. They were incubated with the effector cells (NK and LAK cells) at ratios of effectors to target of 50:1, 25:1, 12:1 and 6:1 in v-bottomed microtiter plates (Costar, Cambridge, MA). After 4 h of incubation at 37°C in 5% $CO_2$, the culture supernatants were harvested and counted in a gamma counter.

Maximal release of the incorporated $^{51}Cr$ into target neuroblastoma cells was measured by lysis of these cells by Triton X-100 (Signam, St Louis, MO). Spontaneous release was measured by counting supernatant of labelled cells incubated in medium only. All testing was done in triplicate. All cell lines were tested for killing by NK and LAK cells twice to four times. Another three volunteer donors were used to ensure that the cytotoxicity seen against neuroblastomas was not unique to the one volunteer donor. Similar ratios of killing between different neuroblastomas were seen with the other volunteer donors, but these experiments were regarded as confirmatory and were not repeated.

The results were expressed as

$$\text{percentage lysed} = \frac{\text{experimental c.p.m.} - \text{spontaneous c.p.m.}}{\text{maximum} - \text{spontaneous chromium release}}$$

Spontaneous release of $^{51}Cr$ ranged from 3 to 20% of maximum.

*Blocking assays*

For the blocking experiments, target cells at $5 \times 10^4\ ml^{-1}$ were incubated with either VLA-4,ICAM-1 (CD54), LFA-1 (CD11a), or LFA-3 (CD58) antibody for 30 min, immediately following incubation with $^{51}Cr$. Three concentrations, 0, 2.5 and 10 $\mu g\ ml^{-1}$, of antibody were used for VLA-4, ICAM-1 and LFA-1, while for LFA-3, the cells were incubated either in medium alone or in the hybridoma supernatant. AD control mouse IgG was added at the same concentration. After incubation, the cells were washed twice. These antibody treated cells were then used as targets for NK and LAK cells as previously.

### Results

NK and LAK cell susceptibility of the neuroblastoma cell lines varied considerably (Figure 1). In all, however, LAK mediated lysis measured in the Cr-release assays was considerably greater than NK killing. All but one neuroblastoma line were strongly positive for GD2 and NCAM, while all were negative for LFA-1 and LFA-2. Expression of LFA-3, ICAM-1, VLA-2, VLA-4 and VLA-6, however, was more variable (shown graphically in Figure 2).

To determine if NK or LAK susceptibility was correlated with expression of any cellular adhesion molecule (CAM) or ligand, percentage killing *vs* CAM/ligand expression was plotted for each line. No correlation can exist between susceptibility to killing and the expression of cell adhesion molecules LFA-1, LFA-2, GD2 or the ligand NCAM, but although levels of VLA 2, 4 and 6 expression varied between cell lines, no correlation was seen between expression of these structures and NK or LAK killing. However, statistically significant correlations were seen between the expression of LFA-3 (the ligand for CD2) and NK and LAK killing (Figures 3a, b). A backward stepwise model was used to assess the ability for each of LFA-3, ICAM-1, VLA-2, VLA-4 and VLA-6 to predict LAK. A backward stepwise model first includes all predictor variables in the model, and in a stepwise fashion deletes one variable at a time until only statistically significant predictor variables remain. The result in the analyses using LAK as the dependent variable was that LFA-3 was the only significant ($P = 0.018$) predictor variable. Now, the supporting evidence for the significance of LFA-3 as a predictor of LAK is that LFA-3 when adjusted for a model containing all other antibodies except VLA-4 remains significant ($P < 0.05$).

Although LFA-3 is a significant predictor variable for NK killing ($P = 0.026$) when considered alone, its predictive

**Figure 1** Shows percentage lysis with the LAK and NK cells as effectors for the different cell lines. Cell lines are ranked in order of the amount of LAK lysis achieved.

**Figure 2** Expression of cell adhesion molecules. LFA-3, ICAM-1, VLA-4, VLA-6 and VLA-2, by the study neuroblastoma lines.

**Figure 3** **a**, Correlation of LFA3 positivity and NK mediated lysis. **b**, Correlation of LFA-3 positivity and LAK mediated lysis.

ability in the presence of other antibodies is diminished. A backward stepwise analysis, similar to that for LAK described above, results in a model containing both ICAM-1 ($P = 0.08$) and VLA-4 ($P = 0.03$). The overall model is significant ($P = 0.04$, $r^2 = 0.55$). These results may depend on the extreme variability (high standard deviation) of the data. Note that there is a significant correlation between LFA-3 and ICAM-1 (Spearman's $r = 0.60$, $P = 0.05$).

As noted, the standard deviation exhibited by each of the antibodies is high. This led to a variance stabilising analysis in which the natural logarithm of both the dependent and independent variables were taken and another regression analysis performed. The result of the backward stepwise regression for LN(LAK) indicates that LFA-3 is significant ($P = 0.04$) even in the presence of (when adjusted for) the four other antibodies. The final model includes only LN(LFA-3) ($P < 0.01$, $r^2 = 0.65$). Performing a similar analysis for LN(NK), LN(LFA-3) is significant when adjusted for all other antibodies except LN(ICAM-1). Again, this is probably due to power. The final model contains only LN(LFA-3) ($P < 0.01$, $r^2 = 0.66$).

To further establish the relevance of CAM expression to cytotoxic vulnerability, neuroblastoma cell lines were incubated with blocking antibodies prior to co-culture with effector cells. No blocking of NK and LAK killing was seen with LFA-1 or VLA-4, but after incubation with ICAM-1, eight out of eleven neuroblastoma lines showed a mean reduction of 30% (range 17–52%) ($t$-test, $P < 0.005$) in LAK killing, at an effector:target ratio of 25:1 (Figure 4). Five out of these eight lines also showed reduction in NK killing with ICAM-1. Blocking was maximal with 2.5 $\mu g\ ml^{-1}$ and not further blocking was seen with 10 $\mu g\ ml^{-1}$. Similarly, LFA-3 reduced LAK killing in seven out of nine lines tested, by a mean of 41% (range 30–76%) ($t$-test, $P < 0.005$) (Figure 5). LFA-3 also reduced NK killing in five of these seven lines. There was no significant blocking with either LFA-1 or VLA-4.

**Figure 4** Percentage reduction in LAK mediated cytotoxicity for eight neuroblastoma cell lines by anti-ICAM-1.

**Figure 5** Percentage reduction in LAK mediated cytotoxicity for seven neuroblastoma lines by anti-LFA-3.

### Discussion

We have shown that the variable sensitivity of neuroblastoma cells to MHC unrestricted killing appears to correlate with variation in their expression of cell adhesion molecules. Cell adhesion molecules are members of the immunoglobulin and integrin supergene families, whose extra-cellular domains participate both in the process of cell adhesion to extra-cellular proteins, and in cell-to-cell adhesion and interaction (Simmons *et al.*, 1988; Kishimoto *et al.*, 1987). These molecules are integral to immune recognition and may play a role in host recognition of transformed cells (Dustin *et al.*, 1987; Makgoba *et al.*, 1989; Sanders *et al.*, 1988). We examined a number of cellular adhesion molecules and their ligands, including those previously described as present on neuroblastoma cells and those known to be important in MHC unrestricted killing (Favrot *et al.*, 1990; Favrot *et al.*, 1991; Gross *et al.*, 1991; Timonen, 1990; Oblakowski *et al.*, 1991).

We found marked differences in the ability of each of these molecules to predict the MHC unrestricted cytotoxic susceptibility of neuroblastoma cells. We confirmed that GD2 and NCAM are commonly and strongly expressed by neuroblastoma cells, so that variation in their expression would not account for differences in cytotoxic susceptibility. The VLA antigens were of greater potential interest. Not only does their expression vary between tumour cell lines, but VLA-4 has been reported to be required for cell-mediated lysis of melanoma, another tumour derived from neural crest cells (Anichini *et al.*, 1990). However, no correlation was found between expression of VLA-4 and susceptibility to NK/LAK lysis and antibody to VLA-4 had no effect on the susceptibility of neuroblastoma lines to cytotoxic effector cells. The VLA receptor ligand system is not therefore of generic importance in interactions of neural crest-derived tumour cells with effector cells of the immune system.

Of all the receptor-ligand systems studied, only LFA-3 (the ligand for LFA-2) and ICAM-1 (the ligand for LFA-1) could be shown to be important in determining susceptibility to MHC unrestricted lysis. Thus, the percentage expression of LFA-3 correlated with cytotoxic susceptibility and the two lines that were least susceptible to killing were both $<1\%$ ICAM-1 positive. The data also imply that only low levels of ICAM-1 expression are required to achieve the maximum susceptibility conferred by this ligand, since it was only at the lowest levels of expression that this molecule appeared to influence the vulnerability of the target cell line.

The importance of LFA-3 and ICAM-1 expression in determining the susceptibility to lysis was confirmed by blocking studies. The majority of the lines tested showing substantial blocking with each antibody. The ability of anti-ICAM-1 monoclonal antibody to block lysis also excludes the possibility that ICAM-1 iteself predicted susceptibility to NK/LAK lysis only because it is co-expressed with LFA-3.

Whether or not this study is relevant to assessing the NK/LAK sensitivity of fresh tumour cells depends on whether ligand expression *in vivo* is similar to that of the neuroblastoma cell lines studied. ICAM-1 expression in clinical specimens at diagnosis has been shown to be restricted to a minority of neuroblastomas. Intriguingly, however, this subset of tumours has a favourable outlook, further supporting the concept that expression of ligands for cell adhesion molecules may determine susceptibility to immune system clearance mechanisms, and improve prognosis. Similarly, LFA-3 was expressed *in vivo* in only a minority of low stage, well differentiated, good prognosis tumours (Favrot *et al.*, 1991). LFA-3 expression can also be induced *in vivo* – even in some high stage undifferentiated tumours – by chemotherapy (Favrot *et al.*, 1991). If LFA-3 and ICAM-1 do indeed confer susceptibility to NK/LAK killing then assessment of tumour susceptibility might most appropriately be made after exposure to chemotherapy and a decision then made as to whether it was appropriate to progress to ABMT and/or therapy with immunostimulators such as IL2. Finally, it will be of interest to discover whether expression of cell adhesion molecules modifies MHC restricted, antigen specific, T cell killing of neuroblastoma cells, if such killing exists (Brenner *et al.*, 1992).

We wish to thank M.L. Hancock, Department of Biostatistics, St Jude Children's Research Hospital, for statistical advice and analysis.

This work was supported in part by NIH grants CA 21765 (CORE), CA 23099 and by the American Lebanese Syrian Associated Charities (ALSAC).

### References

ANDERSON, J.R. & COCCIA, P.F. (1991). Is more better? Dose intensity in neuroblastoma. *J. Clin. Oncol.*, **9**, 902–904.

ANICHINI, A., MORTARINI, R., SPINO, R. & PARIANI, G. (1990). Human melanoma cells with high susceptibility to cell-mediated lysis can be identified on the basis of ICAM-1 phenotype, VLA profile and invasive ability. *Int. J. Cancer*, **46**, 508–513.

BRENNER, M.K., FURMAN, W.L., SANTANA, V.M., BOWMAN, L., MEYER, W., CRIST, W.M., CAMPANA, D., DOUGLASS, E.C., IHLE, J., BOYETT, J., HURWITZ, J., RAO, B.N., JENKINS, J.J., FLETCHER, B., KAUFMAN, W., MOEN, R. & KUEBING, D. (1992). Clinical protocol on Il-2 transduced neuroblastoma. *Human Gene Therapy* (in press).

BRODEUR, G.M., FONG, C.T., MOVITA, M., GRIFFITH, R., HAYES, F.A. & SEEGER, R.C. (1988). Molecular analysis and clinical significance of n-myc amplification and chromosome 1p monosomy in human neuroblastomas. *Prog. Clin. Biol. Res.*, **271**, 3–15.

CAMPANA, D., COUSTAN-SMITH, E. & JANOSSY, G. (1990). The immunological detection of minimal disease in acute leukemia. *Blood*, **76**, 163–171.

D'ANGIO, G.J., EVANS, A.E. & KOOP, C.E. (1991). Special pattern of neuroblasoma with a favorable prognosis. *Lancet*, **1**, 1046–1049.

DUAN, D.S., FARMER, D., RAYNER, A.A. & SADEE, W. (1990). Cytotoxicity of lymphokine-activated killer cells against human neuroblastoma cells: modulation by neuroblast differentiation. *Med. Ped. Oncol.*, **18**, 339–344.

DUSTIN, M.L., SANDERS, M.E., SHAW, S. & SPRINGER, T.A. (1987). Purified lymphocyte function-associated antigen 3 binds to CD2 and mediates T lymphocyte adhesion. *J. Exp. Med.*, **165**, 677.

EVANS, A.E., BAUM, E. & CHARD, R. (1981). Do infants with IVs neuroblastoma need treatment? *Arch. Dis. Child*, **56**, 271–274.

FAVROT, M.C., COMBARET, J.P., WAGNER, E., TABONE, E., BAILLY, C., BOUFFET, E. & PHILIP, T. (1990). Expression of cell adhesion molecules on 45 neuroblastoma samples from 42 patients. *Clin. Chem. Enzym. Comms.*, **2**, 281–285.

FAVROT, M.C., COMBARET, V., GOILLOT, E., TABONE, E., BOUFFET, E., DOLBEAU, D., BOUVIER, R., COZE, C., MICHON, J. & PHILIP, T. (1991). Expression of leucocyte adhesion molecules on 66 clinical neuroblastoma specimens. *Int. J. Cancer*, **44**, 502–510.

GRAHAM POLE, J., CASPER, J., ELFENBEIN, G., GEE, A., GROSS, S., JANSSEN, W., KOCH, P., MARCUS, R., PICK, T., SHUSTER, J., SPRUCE, W., THOMAS, P. & YEAGER, A. (1991). High-dose chemotherapy supported by marrow infusions for advanced neuroblastoma: a Pediatric Oncology Group Study. *J. Clin. Oncol.*, **9**, 152–158.

GROSS, N., BECK, D. & FAVRE, S. (1990). *In vitro* modulation and relationship between N-myc and HLA class I RNA steady-state levels in human neuroblastoma cells. *Cancer Res.*, **50**, 7532–7536.

GROSS, N., CARREL, S., BECK, D. & FAVRE, S. (1991). Cell adhesion molecule expression and modulation in human neuroblastoma cells. In Evans, A.E., D'Angio, G.J., Knudson Jr, A.G. & Seeder, R.C. (eds), *Advances in Neuroblastoma Research*, pp. 293–299, A.R. Liss: New York, NY.

HANDGRETINGER, R., BRUEHELT, G., KIMMING, A., DOPLER, R., NEITHAMMER, D. & TREUNER, J. (1989). *In vitro* induction of lymphokine-activated killer (LAK) activity in patients with neuroblastoma. *Pediatr. Hematol. Oncol.*, **6**, 307–317.

HAYES, F.A. & SMITH, E.I. (1989). Neuroblastoma. In Pizzo, P.A. & Poplack, D.G. (eds), *Pediatric Oncology*, pp. 607–622. J.B. Lippincott: Philadelphia PA.

KISHIMOTO, T.K., O'CONNOR, K., LEE, A., ROBERTS, T.A. & SPRINGER, T.A. (1987). Cloning of the β subunit of the leukocyte adhesion protein: homology to an extracellular receptor defines anovel supergene family. *Cell*, **48**, 681-690.

LOOK, A.T., HAYES, F.A., SHUSTER, J.J., DOUGLASS, E.C., CASTLEBERRY, R.P., BOWMAN, L.C., SMITH, E.I. & BRODEUR, G.M. (1991). Clinical relevance of tumor ploidy and n-myc gene amplification in childhood neuroblastoma: a Pediatric Oncology Group Study. *J. Clin. Oncol.*, **9**, 581-591.

MAIN, E.K., LAMPSON, L.A., HART, M.K., KORNBLUTH, J. & WILSON, D.B. (1985). Human neuroblastoma cell lines are susceptible to lysis by natural killer but not cytotoxic T lymphocytes. *J. Immunol.*, **135**, 242-246.

MAKGOBA, M.W., SANDERS, M.E. & SHAW, S. (1989). The CD2-LFA3 and LFA1-ICAM pathways relevance to T-cell recognition. *Immunol. Today*, **10**, 417-422.

NEGRIER, S., MICHON, J., FLORET, D., BOUFFET, E., GENTET, J.C., PHILIP, I., COCHAT, P., STAMM, D., COSTIL, J., GASPARD, M., ANDREU, G., PALMER, P., FRANKS, C.R., ZUCKER, J.M., BERNARD, J.L., FRIDMAN, W.H., FAVROT, M. & PHILIP, T. (1991). Interleukin-2 and lymphokine-activated killer cells in 15 children with advanced metastatic neuroblastoma. *J. Clin. Oncol.*, **9**, 1363-1370.

NSAR, S., MCKOLANIS, J., PAIS, R., FINDLEY, H., HNATH, R., WALDREP, K. & RAGAB, A.H. (1989). A phase I study of interleukin-2 in children with cancer and evaluation of clinical and immunologic status during therapy. *Cancer*, **64**, 783-788.

OBLAKOWSKI, P., BELLO-FERNANDEZ, C., REITTIE, J.E., HESLOP, H.E., GALATOWICZ, G., VEYS, P., WILKES, S., PRENTICE, H.G., HAZLEHURST, G., HOFFBRAND, A.V. & BRENNER, M.K. (1991). Possible mechanisms of selective killing of myeloid leukaemic blast cells by lymphokine-activated killer cells. *Blood*, **77**, 1996-2001.

PRITCHARD, J., MACELWAIN, T.J. & GRAHAM POLE, J. (1982). High-dose melphalan with autologous marrow for treatment of advanced neuroblastoma. *Br. J. Cancer*, **45**, 86-94.

REITTIE, J.E., GOTTLIEB, D., HESLOP, H.E., LEGER, O., DREXLER, H.G., HAZLEHURST, G., HOFFBRAND, A.V., PRENTICE, H.G. & BRENNER, M.K. (1989). Endogenously generated activated killer cells circulate after autologous and allogeneic marrow transplantation but not after chemotherapy. *Blood*, **73**, 1351-1358.

REYNOLDS, J.V., SHOU, J., CHOI, H., SIGAL, R., ZIEGLER, M.M. & DALY, J.M. (1989). The influence of natural killer cells in neuroblastoma. *Arch. Surg.*, **124**, 235-239.

SANDERS, M.E., MAKGOBA, M.W. & SUSSMAN, E.H. (1988). Molecular pathways of adhesion in spontaneous rosetting of T-lymphocytes to the Hodgkins cell line L428. *Cancer Res.*, **48**, 37-40.

SHUSTER, J.J., CANTOR, A.B., MCWILLIAMS, N., GRAHAM POLE, J., CASTLEBERRY, R.P., MARCUS, R., PICK, T., SMITH, E.I. & HAYES, F.A. (1991). The prognostic significance of autologous bone marrow transplant in advanced neuroblastoma. *J. Clin. Oncol.*, **9**, 1045-1049.

SIMMONS, D., MAKGOBA, M.W. & SEED, B. (1988). ICAM, an adhesion ligand of LFA-1, is homologous to the neural cell adhesion molecule NCAM. *Nature*, **331**, 624-627.

TIMONEN, T. (1990). Characteristics of surface proteins involved in binding and triggering of human natural killer cells. In Schmidt R.E. (ed.) *Natural Killer Cells: Biology and Clinical Application. 6th Int Natural Killer Cell Workshop*, Goslar pp. 18-23. Karger: Basel.